# Provision of HIV testing services and its impact on the HIV positivity rate in the public health sector in KwaZulu-Natal: a ten-year review

**DOI:** 10.1080/17290376.2024.2318797

**Published:** 2024-02-19

**Authors:** Rizwana Desai, Stanley Onwubu, Elizabeth Lutge, Nondumiso Patience Buthelezi, Nirvasha Moodley, Firoza Haffejee, Bontle Segobe, Suresh Babu Naidu Krishna, Maureen Nokuthula Sibiya, Champaklal Chhaganlal Jinabhai

**Affiliations:** a KwaZulu-Natal Department of Health, Health Services Planning, Monitoring & Evaluation, Pietermaritzburg, South Africa; b Chemistry Department, Durban University of Technology, KwaZulu-Natal, South Africa; c School of Nursing and Public Health, University of KwaZulu-Natal, KwaZulu-Natal, South Africa; d Basic Medical Sciences, Durban University of Technology, KwaZulu-Natal, South Africa; e Faculty of Health Sciences, Durban University of Technology, KwaZulu-Natal, South Africa

**Keywords:** HIV testing; HIV testing services; HIV programme; HIV prevention; HIV prevalence; HIV positivity rate; Kwazulu-Natal; DHIS

## Abstract

South Africa has been rated as having the most severe HIV epidemic in the world since it has one of the largest populations of people living with HIV (PLHIV). KwaZulu-Natal (KZN) is the epicentre of the HIV epidemic. The HIV test and treat services in the public health sector are critical to managing the epidemic and responding to the increase in HIV infections. The KwaZulu-Natal Department of Health (DOH) commissioned a review of the provision of HIV testing services in the province and aimed to investigate its impact on the HIV positivity rate over a ten-year period. The study was an ecological study design using data extracted from the Department’s District Health Information System (DHIS). Descriptive analysis was conducted in addition to ANOVA and multiple regression analysis. The results of this study have shown that the total number of HIV tests conducted over the ten-year period in the province has increased with the highest number of HIV tests being conducted in the 2018/2019 MTEF year. ANOVA analysis indicates that there was a statistically significant difference in the total number of HIV tests conducted and the number of HIV tests per 100 000 population across the province’s 11 districts (*p* < 0.001). Statistically significant differences were observed in the HIV testing rate and in the HIV positivity rate over the period (*p* < 0.001). Results from multiple regression analysis showed that the HIV testing rate per 100 000 population was the strongest predictor of the HIV positivity rate. HIV positivity among clients correlated negatively with the number of HIV tests conducted per 100 000 population (r = −0.823; *p* < 0.001) and the HIV testing rate (r = −0.324; *p* < 0.01). This study has found that HIV testing could have an impact on reducing the positivity rate of HIV in the province and is therefore an effective strategy in curbing the HIV epidemic. The KwaZulu-Natal Department of Health should ensure that strategies for implementing and maintaining HIV testing and treating services should continue at an accelerated rate in order to achieve the first 95 of the UNAIDS 2025 SDG target.

## 1. Introduction

South Africa (SA) has been rated as having the most severe HIV epidemic in the world (Kim, Tanser, Tomita, Vandormael, & Cuadros, 2021). The KwaZulu-Natal (KZN) province is reportedly the most affected province in the country and is commonly referred to as the epicentre of the HIV epidemic (Kim et al., 2021; Bello, 2019). Hence, studies have recommended that the KZN province should concentrate its efforts on the provision of HIV services in order to curb to HIV epidemic (Kim et al., 2021).

One of the goals of South Africa’s National Strategic Plan (NSP) for HIV, TB and STIs 2017–2022 is to address the burden of HIV in the country through the use of strategic information such as routinely collected data for monitoring the progress towards achieving the United Nations (UN) Sustainable Development Goals (SDGs) as well as to determine the utilisation of services (National Department of Health, 2016c). There is also a need to evaluate the impact of interventions on programmes that are implemented in response to the burden of disease and to ensure that interventions are focused on high-burden areas with populations that are most at risk (Wabiri, Naidoo, Mungai, Samuel, & Ngwenya, 2019). Routinely collected data from public health facilities are housed on the District Health Information System (DHIS). The aggregated data from DHIS can be used for epidemiological monitoring as well as estimating the burden of HIV at various geographic levels (Wabiri et al., 2019).

One of the recommendations emanating from the 2016/2017 District Health Barometer (DHB), which is an annual publication published by the Health Systems Trust (HST) that provides district managers with strategic information by analysing amongst others, DHIS data, was that the ‘HIV testing rate should be cross-tabulated with the HIV positivity rate to assess whether HIV testing improves HIV case finding’ (Molapo, Schultz, Sello, & Mawela, 2017). Three key indicators that are part of the routinely collected data that can be found on the DHIS that are directly linked to the DHB recommendation are: the total numbers of HIV tests conducted at public health facilities, the HIV testing rate and the HIV positivity rate. The total number of HIV tests conducted provides insight into whether HIV/AIDS is being combatted and the burden of HIV is decreasing. HIV testing services (HTS) are known as the ‘the gateway to a complete continuum of care’ since it plays a critical role in accessing other preventative services offered by the Department of Health (Maheu-Giroux et al., 2019; National Department of Health, 2016b). The HIV testing rate can be used to assess the acceptability and uptake of HIV testing by clients as well as a proxy to determine the extent to which the first 95 of the 95-95-95 UNAIDS 2025 target is being achieved (KwaZulu-Natal Office of the Premier HIV/AIDS Directorate, 2017). The rate of HIV infections within the public health system in KZN can be continuously monitored using an indicator called the HIV positivity rate which represents the annual proportion of clients at public health facilities who tested positive for the first time in the 15–49 years age category from amongst the total number of 15–49 year old clients that underwent HIV testing. This excluded antenatal clients (ANC) who were found to be HIV positive after undergoing HIV testing (Wabiri et al., 2019).

Although several studies have examined HIV testing services and the trends in the HIV positivity rate in KZN, these studies have been conducted mainly at either the patient level, facility level or community level. None have examined HIV testing services and its impact on the HIV positivity rate at the provincial and district level over a long period of time.

This study aims to assess the impact of the KZN Department of Health’s HIV testing programme on the HIV positivity rate in KwaZulu-Natal on a population level. The objectives of the study are to determine the trends in the total number of test conducted annually, the rate of HIV tests conducted per 100 000 population in order to assess whether HIV testing is proportionate to the population size, the HIV testing rate, the HIV positivity rate and to determine whether HIV testing services have impacted on the HIV positivity rate in the KZN province and its districts over a ten-year study period.

## 2. Methodology

The KZN province which is found on the south-eastern part of South Africa was the setting for this study (Municipalities of South Africa, 2020). It is the second most populated province in the country with almost 20% of the country’s population of 11.3 million people in 2019 (KwaZulu-Natal Provincial Government, 2017, 2021). The life expectancy in both males and females in KZN in 2020 was lower than the national level (KwaZulu-Natal Provincial Government, 2021). This province has one metropolitan district municipality, eThekwini, 10 district municipalities and 43 sub-district municipalities (Municipalities of South Africa, 2020). Across the province, there are 72 public sector hospitals, 589 primary healthcare clinics (PHCs) and 22 community health centres (CHCs). Approximately 88% of the provincial population does not have medical insurance (KwaZulu-Natal Department of Health, 2021).

An ecological study design was employed to analyse aggregated secondary data at the KZN provincial level which is housed on the electronic routine health information system called the web District Health Information System (webDHIS) Version 2.30. WebDHIS is a web-based health information management tool that is used to collect, validate, analyse, and present aggregate data for routine reporting purposes. The software was first rolled out in 1999 with the introduction of a minimum data set at the time, collecting just a few essential data elements throughout the country. This version of the DHIS was implemented as a standalone system at each facility, storing all data pertaining to that particular facility, with no link to other facilities or levels of management. In 2016, an advancement to the DHIS was introduced, which is called the DHIS 2, and is most commonly known as the webDHIS. One of the major improvements when comparing the old system to the new was that it allowed data to be available almost immediately at all levels of administration making it easier for verification and reporting of data.

The transition from the access-based DHIS to the web-based version was introduced in a phased approach, which was concluded by the end of March 2017. All previously collected data was moved from the Microsoft Access version and migrated to the new central database. The Department relied on trend analysis over a period of a few years to confirm if the data was migrated correctly. Following this quality check, some inconsistencies were identified on how data was migrated and merged, but these were resolved over a few months. Data capture and reporting reached stability during the 2017 financial year. During this analysis, data was extracted from the webDHIS only, which now houses data prior to the 2016 conversion as well as the newly captured data directly onto the system.

In the current process flow for data management in KZN, data is collected for each health facility using a selected list of health-related indicators that are prescribed by the National and Provincial Department of Health (KwaZulu-Natal Department of Health, 2018). Routinely collected data are collected on paper within the health facility and electronically captured by information officers. The health facility data is reported to the district on a monthly basis, which is then aggregated at the provincial level. The web based system allows captured information at the facility level to be collated at the district, provincial and national levels within 24 hours (KwaZulu-Natal Department of Health, 2018). The collection of indicators that was developed by the National Department of Health (NDoH) for purposes of monitoring the performance of the healthcare system on the DHIS is called the National Indicator Dataset (NIDS). The Provincial Indicator Dataset, PIDS, is a specific set of indicators that is province specific (Department of Health, 2011). Data collection tools at public health facilities have been aligned in accordance to the NIDS, (2017), whilst the PIDS have been compiled in consultation with Programme Managers (KwaZulu-Natal Department of Health, 2018). WebDHIS was rolled out in December 2016 in hospitals and CHCs. Since aggregated data was used, all public sector health facilities in KZN were included in this study and there was no sampling employed.

Data was mined from the webDHIS database for the following indicators: the total number of HIV tests conducted, the rate of HIV testing, the HIV positivity rate and the population. The source of the former indicator were registers at PHC’s and HIV Testing Services (HTS) Register or the HCT module in TIER.Net (KwaZulu-Natal Department of Health, 2021). According to the NIDS, the definitions of the indicators analysed for this study can be found in Table A1 in Appendices section**.** The indicator, ‘total number of HIV tests conducted’, was used to calculate the rate per 100 000 population for the province and for each district within the province in order to take into account the effect of population variation for comparisons amongst districts. The study period that was under review was according to the Mid Term Expenditure Framework (MTEF) Years between the 01st of April 2009 and the 31st of March 2019. Data for the province and the 11 health districts were extracted from webDHIS. Data was then exported to a Microsoft Excel workbook for descriptive analysis and time trend analysis. Statistical analysis was performed using the statistical software package, Statistical Package for Social Sciences (SPSS), Version number 26. The mean and standard deviation for the dataset were calculated. Analytical data analysis was limited to district level data and excluded provincial level data due to small variation in the dataset. The difference in means between districts was determined by performing a one-way ANOVA test. The Bonferroni post hoc test was run to determine which specific districts differed from each other using a 95% confidence interval (CI) (*p* ≤ 0.05). The percentage change in the total number of HIV tests conducted in the KZN province was calculated by subtracting the previous year’s number from the recent year’s number and dividing by the previous number and converting it into a percent.

Ethical approval for the study was granted by the University of KwaZulu-Natal Biomedical Research Ethics Committee, BREC (BCA056/13). Patient consent was not required since this study used aggregated data without any patient identifiers. The aggregate data consists of counts for data elements across health facilities in the province.

## 3. Results

Results are shown for the number of HIV tests conducted (in all age groups), the number of HIV tests conducted per 100 000 population, the HIV testing rate (excluding antenatal clients) and the positivity rate of HIV (excluding antenatal clients) at the provincial and district level over the ten-year review period (2009/2010 till 2018/2019).

### 3.1. Total number of HIV tests conducted in the KwaZulu-Natal (KZN) province

**Figure 1.**
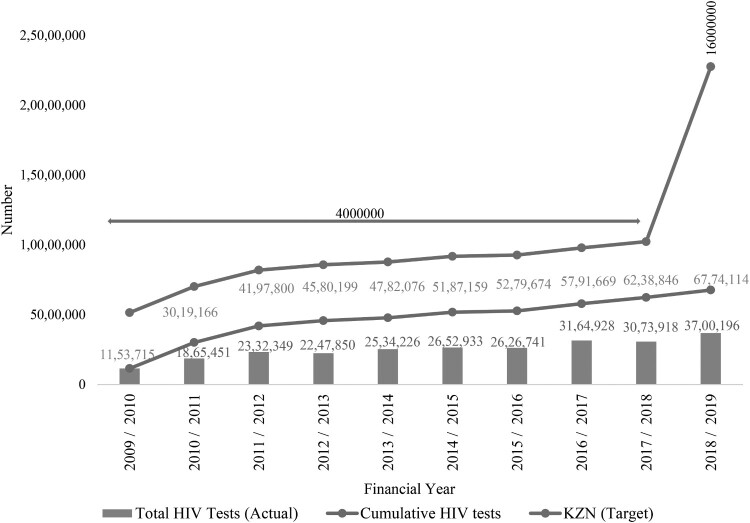
Total number of HIV tests conducted, the cumulative number of HIV tests and the KZN province’s target for the number of HIV tests between 2009/2010 and 2018/2019.

The trend in the number of HIV tests conducted between 2009/2010 and 2018/2019 MTEF years in the KwaZulu-Natal (KZN) public health sector is shown in Figure 1.

#### 3.1.1. Total number of HIV tests conducted in KZN

Figure 1 provides an overview of the HIV testing coverage and the target for HIV testing for the province during the period 2009/2010–2018/2019. The peak point in 2018/2019 indicates the target set out by the Department of Health (DOH) to conduct approximately 16.5 million HIV tests cumulatively in KZN by March 2020. The mean number of actual HIV tests conducted over the ten-year period in the province was 2 535 230.70 (SD = 710 791.03). The above figure illustrates the increase in the number of HIV tests conducted from the 2016/2017 MTEF year onwards (N = 3 164 928) compared to the initial HIV testing number in 2009/2010 (N = 1 153 715). This amounted to a 174% increase in HIV testing services in KZN. The highest number of HIV tests conducted in the province was in the 2018/2019 MTEF year (N = 3 700 196). A similar trend can be seen for the cumulative number of HIV tests conducted over the ten-year period in the province.

Between the 1^st^ April 2009 and March 31st, 2020, a cumulative number of 6 774 114 HIV tests were conducted throughout the province (Figure 1). The peak point in 2018/2019 indicated by the red line in the above figure, is the target set out by the Department of Health which was to conduct approximately 16.5 million HIV tests cumulatively in KZN by March 2020. Even though there was a noticeable increase in the number of HIV tests conducted in the province, the KZN DOH was still far from achieving its target.

#### 3.1.2. Total number of HIV tests conducted at districts in KZN

Table 1 indicates the total number of HIV tests conducted at districts in KZN. The eThekwini district, which is considered a metropolitan district in the province, conducted the highest number of HIV tests with a mean of 763 390 (SD = 296973.57) over the study period whilst the uMkhanyakude district conducted the lowest number of HIV tests (M = 137 063). ANOVA analysis revealed that there was a statistically significant difference in the numbers of HIV tests conducted between the 11 districts in the province (*p* < 0.001). The Bonferroni test suggests that the total number of HIV tests conducted in the eThekwini district were significantly higher than those measured for the other 10 districts (*p* < 0.001).

**Table 1.** Mean, standard deviation, ANOVA and Bonferroni post hoc analysis of the total number of HIV tests conducted at the 11 districts in the province between 2009/2010 and 2018/2019.

District	Total number of HIV tests conducted per district			
	Mean	SD	*p* value	Post hoc
Amajuba^1^	138 828.60	32 943.59	0.000	0.000^2,1^
eThekwini^2^	763 390.70	29 6973.57		1.00
Harry Gwala^3^	153 026.70	34 533.56		0.000^2,3^
iLembe^4^	139 244.80	28 793.29		0.000^2,4^
King Cetshwayo^5^	231 157.60	63 937.91		0.000^2,5^
Ugu^6^	221 972.20	62 804.75		0.000^2,6^
uMgungundlovu^7^	232 962.10	71 422.96		0.000^2,7^
uMkhanyakude^8^	137 063.00	32 541.54		0.000^2,8^
uMzinyathi^9^	169 499.30	57 366.70		0.000^2,9^
uThukela^10^	139 485.50	29 116.29		0.000^2,10^
Zululand^11^	208 600.20	45 263.45		0.000^2,11^

Abbreviation: SD, standard deviation.

Note: Superscript numbers indicate significant differences between the sample groups (ANOVA, *p *< 0.05)

#### 3.1.3. Total number of HIV tests conducted during the study period at districts in KZN

The mean, standard deviation, and ANOVA value measured for the total number of HIV tests conducted in the KwaZulu-Natal province for the study period are provided in Table 2.

**Table 2.** ANOVA test comparison for the total number of HIV tests conducted at the 11 districts in KZN by the study period.

Period	Total number of HIV tests conducted		
	Mean	SD	*p* value
2009/2010	104 883.18	66 477.740	0.312
2010/2011	169 586.45	85 055.516	
2011/2012	212 031.73	152 211.932	
2012/2013	204 350.00	142 105.602	
2013/2014	230 384.18	154 648.877	
2014/2015	241 175.73	176 218.367	
2015/2016	238 794.64	215 329.589	
2016/2017	287 720.73	264 068.511	
2017/2018	279 447.09	252 583.223	
2018/2019	336 381.45	311 918.121	

Abbreviation: SD, standard deviation.

Even though there was an increase in the number of HIV tests conducted over the study period, there was no significant difference in the total number of HIV tests conducted between the financial years over the ten-year review period at districts (*p* > 0.05).

### 3.2. The rate of HIV tests conducted per 100 000 population in the KwaZulu-Natal (KZN) province

The mean rate of HIV tests being conducted per 100 000 population for the province during the study period was 23 329.80 per 100 000 (SD = 5669.90). This was below the annual target that was required to achieve the cumulative target of conducting 16.5 million HIV tests in KZN by March 2020.

In Table 3, the ANOVA value indicates that there was a statistically significant difference for the number of HIV tests conducted per 100 000 population between the 11 districts (*p* < 0.001).

**Table 3.** Mean and standard deviation of the number of tests per 100 000 population between the 11 districts in the province over the ten-year study period.

District	HIV tests conducted (per 100 000 population) per district			
	Mean	SD	*p* value	Post hoc
Amajuba^1^	25 685.27	5467.831	0.000	1.00
eThekwini^2^	21 125.44	7523.789		1.00
Harry Gwala^3^	31 539.20	6001.316		0.012^2,3^
iLembe^4^	21 134.05	3569.086		0.012^3,4^
King Cetshwayo^5^	24 291.59	6119.158		1.00
Ugu^6^	29 705.26	7364.475		0.014^6,10^
uMgungundlovu^7^	21 417.88	5538.069		0.018^3,7^
uMkhanyakude^8^	20 767.43	4320.843		0.018^8,9^
uMzinyathi^9^	30 900.67	9665.775		0.040^7,9^
uThukela^10^	19 420.70	3984.774		0.003^9,10^
Zululand^11^	25 129.47	4497.421		1.000

Abbreviation: SD, standard deviation

Note: Superscript numbers indicate significant differences between the sample groups (ANOVA, *p *< 0.05)

The above table is an indication of the rate of HIV tests being conducted at districts in proportion to its population size. The results found that districts with smaller population sizes such as the Harry Gwala district and the uMzinyathi district had significantly higher proportion of HIV tests being conducted per 100 000 population (*M *= 31 539, SD = 6001.32 and *M* = 30 900, SD = 9665.775) respectively, than the larger districts in the province such as the eThekwini (*M* = 21 125.44, SD = 7523.789) and uMgungundlovu districts (*M* = 21 417.88, SD = 5538.069). This was confirmed by the Bonferroni test (*p* < 0.001).

#### 3.2.1. The HIV testing rate per 100 000 population over the study period at districts in KZN

Table 4 shows the HIV testing rate per 100 000 population over the study period at districts in KZN. The highest mean value measured for the HIV testing rate per 100 000 population over the study period at districts in KZN was found to be during the period 2018/2019 (*M* = 32 480) while the lowest was for the period 2009/2010 (*M* = 12 650). ANOVA analysis indicated a statistically significant difference in the number of HIV tests conducted per 100 000 population (*p* < 0.001). The results from the Bonferroni test suggests that the total number of HIV tests conducted for the period 2018/2019 was significantly higher than the initial period when HIV testing began in 2009/2010 (*p* < 0.001).

**Table 4.** ANOVA test comparison for the number of HIV tests conducted per 100 000 population between the 11 Districts in KZN over the ten-year period

Period	Number of HIV tests conducted (per 100 000 population)			
	Mean	SD	*p* value	Post hoc
2009/2010^1^	12 650.38	2767.927	0.000	0.000^10,1^
2010/2011^2^	20 984.14	4878.613		0.000^10,2^
2011/2012^3^	24 243.05	3540.622		0.020^10,3^
2012/2013^4^	23 290.44	4663.849		0.004^10,4^
2013/2014^5^	26 170.98	5615.794		0.286^10,5^
2014/2015^6^	26 309.45	5012.362		0.340^10,6^
2015/2016^7^	24 265.38	5302.994		0.020^10,7^
2016/2017^8^	28 650.95	7439.715		1.000^10,8^
2017/2018^9^	27 424.37	5429.319		1.000^10,9^
2018/2019^10^	32 480.82	6810.686		1.000

Abbreviation: SD, standard deviation.

Note: Superscript numbers indicate significant differences between the sample groups (ANOVA, *p *< 0.05).

### 3.3. The HIV testing rate in the KwaZulu-Natal (KZN) province

#### 3.3.1. The HIV testing rate in KZN

The mean HIV testing rate for the province during the ten-year period was 96.30% (SD = 8.83). The lowest testing rate was 81% during the initial period when HIV testing commenced, during the 2010/2011 MTEF year whilst the maximum testing rate (116%) was during the 2016/2017 MTEF year.^1^ The high rate in the 2016/2017 MTEF year maybe due to a system error during the conversion of DHIS to webDHIS during December 2016.

#### 3.3.2. The HIV testing rate at districts in KZN

The data in Table 5 shows the HIV testing rate at districts in KZN. The proportion of clients undergoing HIV tests from amongst those that were counselled for HIV testing was lower in the metropolitan district of the province, the eThekwini district (*M* = 90.7, SD = 12.0) compared to the other districts. The Zululand district was found to have the highest mean HIV testing rate (*M* = 103.4, SD = 21.2) over the ten-year period. However, the ANOVA analysis revealed that there was no real difference in the HIV testing rate between districts (*p* > 0.05).

**Table 5.** ANOVA test comparison for the HIV testing rate at the 11 Districts in KZN.

District	HIV testing rate per district		
	Mean	SD	*p* value
Amajuba	99.9	4.7	0.337
eThekwini	90.7	12.0	
Harry Gwala	96.3	3.4	
iLembe	102.3	13.3	
King Cetshwayo	94.1	16.8	
Ugu	100.6^3^	9.1	
uMgungundlovu	103.0	16.8	
uMkhanyakude	99.1	2.3	
uMzinyathi	96.2	3.8	
uThukela	98.7	5.0	
Zululand	103.4	21.2	

Abbreviation: SD, standard deviation.

#### 3.3.3. The HIV testing rate over the study period at districts in KZN

The table below (Table 6) illustrates the HIV testing rate at districts over the ten-year study period.

**Table 6.** ANOVA test comparison for the HIV testing rate at the 11 Districts in KZN by the study period.

Period	HIV testing rate			
	Mean	SD	*p* value	Post hoc
2009/2010^1^	94.1	2.6	0.000	0.000^1,8^
2010/2011^2^	88.41	13.9		0.000^1,2^
2011/2012^3^	93.7	7.2		0.000^1,3^
2012/2013^4^	96.4	5.9		0.000^1,4^
2013/2014^5^	97.5	1.7		0.000^1,5^
2014/2015^6^	98.8	1.1		0.000^1,6^
2015/2016^7^	98.9	1.8		0.000^1,7^
2016/2017^8^	120.4	22.5		0.000^1,8^
2017/2018^9^	98.9	1.7		0.000^1,9^
2018/2019^10^	98.4	2.7		0.000^1,10^

Abbreviation: SD, standard deviation.

Note: Superscript numbers indicate significant differences between the sample groups (ANOVA, *p *< 0.05).

ANOVA analysis revealed that the HIV testing rate was significantly different between the various financial years under review (*p* < 0.001). The highest mean HIV testing rate measured was for the period 2016/2017 (*M* = 120.4) while the lowest HIV testing rate was found to be during the commencement of HIV testing services by government during the period, 2009/2010 (*M* = 94.1). The Bonferroni test suggests that the testing rate for the period 2016/2017 was significantly higher than any other periods (*p* < 0.001).

### 3.4. The HIV positivity rate in the KwaZulu-Natal (KZN) province

#### 3.4.1. HIV positivity rate in KZN

**Figure 2.**
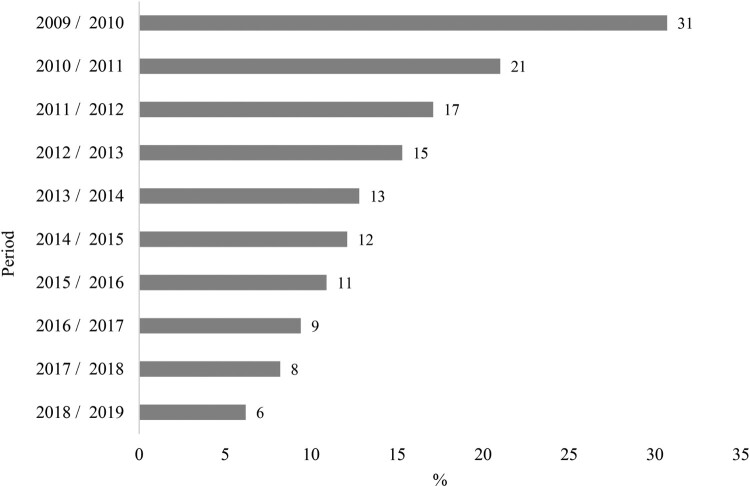
HIV positivity rate of 15–49 year old in the public sector in KZN between 2009/2010 and 2018/2019.

The mean HIV positivity rate of clients between the ages of 15 and 49 years old for the province over the ten-year study period was 14% (SD = 7). Figure 2 shows the highest HIV positivity rate of 31% was in the 2009/2008 MTEF year, when HIV testing services first began whilst the lowest HIV positivity rate in the province was 6% during the 2018/2019 MTEF year, when efforts to increase HIV prevention was augmented.

#### 3.4.2. HIV positivity rate per district in KZN

Analysis of the HIV positivity rate which is an indicator of the HIV infection rate, reveals that the larger populated districts such as the eThekwini and uMgungundlovu districts had higher HIV positivity rates (*M *= 18.8 and *M* = 16.3) respectively compared to the smaller districts, Harry Gwala and uMzinyathi districts (*M* = 9.7) which showed the lowest HIV positivity rates in the province over the study period (Table 7). However, the ANOVA values indicated that there was no significant difference in the positivity rate of HIV between the 11 districts (*p* > 0.05).

**Table 7.** Mean and standard deviation of the HIV positivity rate at the 11 districts in the province over the ten – year study period.

District	HIV positivity rate per district (%)		
	Mean	SD	*p* value
Amajuba^1^	12.4	6.4	0.143
eThekwini^2^	18.8	9.6	
Harry Gwala^3^	9.7	5.5	
iLembe^4^	13.9	5.2	
King Cetshwayo^5^	13.9	6.7	
Ugu^6^	11.7	6.3	
uMgungundlovu^7^	16.3	7.8	
uMkhanyakude^8^	12.6	6.4	
uMzinyathi^9^	9.7	7.8	
uThukela^10^	15.0	7.4	
Zululand^11^	12.2	6.2	

Abbreviation: SD, standard deviation.

Note: Superscript numbers indicate significant differences between the sample groups (ANOVA, *p *< 0.05).

#### 3.4.3. The positivity rate of HIV over the study period in KZN

When measuring the HIV infection rate in the province over the study period, it was found that the highest HIV positivity rate was measured for the period 2009/2010 (*M* = 28.9) while the lowest (*M *= 5.8) was observed in the 2018/2019 period (Table 8). The Bonferroni test suggests the HIV positivity rate measured in the 2009/2010 MTEF was significantly higher when compared to any other period (*p* < 0.001).

**Table 8.** Mean and standard deviation of the HIV positivity rate at the 11 districts in the province over the ten-year study period.

Period	HIV positivity rates			
	Mean	SD	*p* value	Post hoc
2009/2010^1^	28.9	4.5	0.000	0.000^8,1^
2010/2011^2^	19.8	4.0		0.000^8,2^
2011/2012^3^	15.5	3.5		0.000^8,3^
2012/2013^4^	13.8	3.6		0.015^8,4^
2013/2014^5^	11.5	3.4		1.000^8,5^
2014/2015^6^	11.0	2.6		1.000^8,6^
2015/2016^7^	10.1	2.3		1.000^8,7^
2016/2017^8^	8.9	2.2		1.000
2017/2018^9^	7.7	1.8		1.000^8,9^
2018/2019^10^	5.8	1.5		0.929^8,10^

Abbreviation: SD, standard deviation.

Note: Superscript numbers indicate significant differences between the sample groups (ANOVA, *p *< 0.05).

### 3.5. The impact of the number of HIV tests that were conducted, the rate of HIV tests conducted per 100 000 population and the HIV testing rate on the positivity rate of HIV in the KwaZulu-Natal (KZN) province

In Table 9, the HIV positivity rate among clients correlates negatively with the total number of HIV tests conducted per 100 000 population (r = −0.823; *p* < 0.01), and the HIV testing rate (r = −0.324; *p* < 0.01). Hence, the higher the number of HIV tests being conducted per 100 000 population and the higher HIV the testing rate, the lower the positivity rate of HIV. No association was found between the total number of HIV tests conducted and the HIV positivity rate among clients.

**Table 9.** Pearson correlation between the HIV positivity rate and its predictors.

		Total number of HIV tests conducted	Number of HIV tests conducted per 100 000 population	HIV positivity rate	HIV testing rate
Total number of HIV tests conducted	Pearson correlation	1	.173	-.102	-.033
	Sig. (2-tailed)		.070	.287	.729
	N	110	110	110	110
Total number of HIV tests conducted per 100 000 population	Pearson correlation	.173	1	-.823**	.212*
	Sig. (2-tailed)	.070		.000	.027
	N	110	110	110	110
HIV positivity rate	Pearson correlation	–.102	–.823**	1	-.324**
	Sig. (2-tailed)	.287	.000		.001
	N	110	110	110	110
HIV testing rate	Pearson correlation	–033	.212*	-.324**	1
	Sig. (2-tailed)	.729	.027	.001	
	N	110	110	110	110

**correlation is significant at the 0.01 level (2-tailed).

## 4. Discussion

In order to ensure that the government is adequately making an impact on the burden of disease when implementing programmatic interventions, it is crucial that these programmes are evaluated. The findings from this study provide an overview of whether HIV testing services provided by the KZN Department of Health has made an impact on the HIV infection rate over the study period of the KZN population which is the most severely affected by HIV.

### 4.1. HIV testing in the KwaZulu-Natal (KZN) province

A drastic increase in HIV testing in the province in the last four financial years was observed compared to when HIV testing services were first introduced, and this can be attributed to various governmental initiatives that were implemented to increase prevention of HIV infections. During the 2015/2016 MTEF, the South African government commenced implementing the 90-90-90 HIV, AIDS and TB strategy and have now adopted the UNAIDS 95-95-95 strategy to achieve HIV epidemic control by 2030 together with initiatives such as Provider Initiated Counselling and Test (PICT). The KZN Department of Health (KZN DoH) itself initiated campaigns to reduce the incidence of HIV such as the Hlola Manje Zivikele at districts; ‘Test for HIV at least once a year’ media campaigns and the ‘First things First’ counselling and testing campaign during the 2015/2016 year which would have had an impact on the 2016/2017 MTEF (KZN Department of Health, 2016).

A significant reason for the increase in the total number of HIV tests conducted in 2016/2017 MTEF may be due to the ‘Universal Test and Treat’ (UTT) approach that was recommended by the World Health Organisation (WHO) in 2016 and adopted by South Africa in the same year (Department of Health (SA), 2016; National Department of Health, 2016a). During 2016, the National Department of Health also implemented the HIV Testing Services (HTS) Policy which included HIV self-testing (HIVST) as one of the interventions for expanding HTS (National Department of Health, 2016b; Venter et al., 2017). The HEAids (Higher Education HIV and AIDS) Programme in partnership with higher learning institutions was also expanded during this particular year (KZN Department of Health, 2017). In addition, other provincial wide prevention programmes to reduce HIV infections continued through the 2016/2017 MTEF year such as the Unfinished Business Project, the DREAMS project, an initiative targeting adolescent girls and young women, and the She Conquers programme which targeted young women and girls (KwaZulu-Natal Department of Health, 2018). The increase in HIV testing in the 2017/2018 MTEF year could be attributed to the data itself. In 2017, the NIDS on DHIS was amended and the definition of the total number of HIV tests conducted was redefined to include antenatal care (ANC) clients (Department of Health, 2019). The number of HIV tests conducted on ANC clients are conducted twice during pregnancy, the first is at presentation (prior to 20 weeks), and the second is at approximately 32 weeks. According to the KZN Department of Health’s Annual Report, (2017), the KZN Department of Health intended targeted testing of those individuals that are unaware of their HIV status at the community and population level in the 2018/2019 MTEF year (KZN Department of Health, 2017). In 2018, the South African President, Mr Ramaphosa, introduced a multi-sectoral national wellness campaign at his State of the Nation address. This campaign entailed conducting HIV tests on a minimum of 10 million people per annum nationally from 2018 to 2020 (Massyn, Pillay, & Padarath, 2019). This coincides with the higher number of HIV tests conducted in KZN between 2018 and 2019 compared to previous reporting periods. Therefore, the substantial increase in the number of HIV tests conducted in public health facilities in KZN and its districts is mainly due to the national and provincial Department of Health’s rigorous determination in expanding and strengthening HIV testing services through the various policies and interventions that were introduced and implemented.

### 4.2. HIV testing at districts

This study revealed that the two larger districts in the province, the eThekwini district and the uMgungundlovu district conducted the most number of HIV tests over the review period respectively, and this may be due to the province identifying these two districts in their provincial plan as the focus for intensified HIV prevention interventions known as the ‘Focus for Impact’ methodology (Kalonji & Mahomed, 2019; KwaZulu-Natal Office of the Premier HIV/AIDS Directorate, 2017). According to the Multi-Sectoral Response Plan for HIV, TB and STIs for the KwaZulu-Natal Province, 2017-2022, this methodology directs prevention of HIV infection intervention strategies to areas that show the highest disease burden (Kalonji & Mahomed, 2019; KwaZulu-Natal Office of the Premier HIV/AIDS Directorate, 2017).

Study results found geographic variation between urban and rural districts and population density that may account for the difference in the number of tests conducted per 100 000. The effect of area population size between urban and rural areas as implied by Wabiri et al. (2019), may account for the discrepancy in the rate of HIV tests being conducted per 100 000 population and the HIV testing rates (Wabiri et al., 2019). Therefore, in order to achieve ideal HIV testing coverage, it is necessary to provide more equitable geographic distribution of HIV testing services. The variation in HIV testing rate at districts may point to the quality of HTS and the uptake of HIV testing services. Even though there are large number of tests being conducted at larger districts, in proportion to the size of the districts, HIV testing services may not be accessible to the entire population of the larger districts. The uptake of HIV testing services and methods to encourage testing amongst the male population and other hard to reach populations in mainly urban districts needs to be one of the focal strategies for improving HIV testing in the two larger districts. Other methods of HIV testing need to be included in the provision of HIV testing services by the KZN DoH at public health facilities such as HIV self-testing and index testing or partner-assisted notification which is recommended by the World Health Organisation (WHO). In addition, the KZN DoH could also expand HIV testing services to include community-based HIV testing services such as home-based HIV self-testing, which may also assist in providing services to ‘key populations’ such as sex workers, men having sex with men (MSM), people who inject drugs and transgender populations (van Schalkwyk et al., 2021).

### 4.3. HIV infection rates in the kwazulu-Natal (KZN) province

According to van Schalkwyk et al. (2021), geospatial variation in the prevalence of HIV is accountable for distribution of HIV epidemic in South Africa (van Schalkwyk et al., 2021). The results of this study indicate that the HIV positivity rate in KZN is declining over the past few years. These results are comparable to the national results described in the 2018 Global AIDS Monitoring Report, which found that the HIV positivity rate in South Africa was 8.3%, 7.4% and 6.6% in 2016, 2017 and 2018 respectively (Bello, 2019). This study identified the two larger districts, eThekwini and uMgungundlovu, as having a high HIV positivity rate over the 10-year review period. These districts were identified as HIV ‘hotspots’ in the 2015/2016 MTEF in a study by Wabiri et al., who used the same indicator for analysis (Wabiri et al., 2019). These districts were also identified in the NSP for HIV, TB and STI’s, 2017 −2022, as districts that contributed to the high national HIV prevalence rates (National Department of Health, 2016c). van Schalkwyk et al. (2021), found that the eThekwini district had the highest number of people living with HIV in 2018 compared to other metropolitan districts (van Schalkwyk et al., 2021). The eThekwini district also has the majority share of the population of 33% even though the land area is only 2.4% (KwaZulu-Natal Office of the Premier HIV/AIDS Directorate, 2017). Therefore, this study also agrees with the study results of Wabiri et al. (2019), that areas displaying higher rates of HIV infection are areas of high population density (Wabiri et al., 2019). The plausible reason for this may be attributed to the fact that cities often attract a large influx of migrants, and this migration phenomenon is closely linked to an increased risk of HIV infection (Welz et al., 2007). The urban population tends to have a higher proportion of males, which is associated with elevated levels of commercial sex activities that can contribute to the transmission of HIV (South, Trent, & Bose, 2012).

The study results indicate that HIV testing which is part of the HIV prevention programme is effective in controlling the HIV epidemic in the public sector in KwaZulu-Natal. This study confirms that the response by the Provincial government to focus their HIV prevention efforts in areas with high HIV burdens is appropriate. However, it is recommended that the range of HIV testing in the provision of HIV testing services is expanded by the KZN DoH in order to improve HIV testing coverage in most especially larger districts in order to achieve equitable geographic distribution of HIV testing services.

## 5. Conclusion

This study has described HIV testing services within the province and the districts within the KZN province. The number of HIV tests conducted in the province has been increasing over the study period and this is due to government efforts in introducing policies and interventions. However, greater efforts are needed by the province to achieve its target of ensuring that 95% of all people living with HIV know their HIV status by increasing HIV testing services. Districts in KZN with high HIV positivity rates, low number of tests per 100 000 population and low HIV testing rates should be the focus of interventions for increasing HIV testing. Greater coverage of HTS per population density is required. Continuous monitoring of the HIV indicators used in this study should be assessed in order to provide a strategic response to reducing HIV infections in an HIV endemic population. In order to increase efforts in achieving the UNAIDS 95-95-95 targets of eliminating the HIV epidemic by 2030, greater efforts to test all patients accessing public health services and taking services to the community and household level should be encouraged.

## 6. Recommendations

Continued efforts to reduce the spread of HIV by the Department of Health are required. Further studies on the utilisation of HIV testing services in the public health sector using sociodemographic factors should be commissioned. The use of the health patient registration system (HPRS) will provide a deeper understanding of the utilization patterns of HIV testing services by various population groups in order to provide specific tailor-made interventions. The impact of the COVID-19 pandemic on HIV testing services could also be measured and an evaluation of the HIV index testing services should be undertaken. Further examination into the geographic variations of the HIV epidemic, the quality and type of HTS services being offered, resource allocation of HTS services and barriers to uptake of public health services between districts would be beneficial to the Department’s response in reducing HIV infections amongst the KZN population.

## 7. Limitations of study

The study used an ecological study design utilising a secondary data source which was data housed on the DHIS. Hence, the results cannot be generalized to the population since aggregated data was used for analysis but is limited to those accessing the public health sector. Secondary data sources may also be prone to system errors.

## Data Availability

Data from the study can be accessed from the KwaZulu-Natal Department of Health’s Data Management and Geographic Information System Unit by completing a Data user agreement form.

## Notes

1 This is according to DHIS data.

3 Some Districts may show a rate of greater than 100% due to capturing errors. However, they have been included in the dataset since this is audited data. Abbreviation: SD, standard deviation

## Appendices

**Table A1.** Definition of indicators extracted from webDHIS according to the National Indicator Dataset (NIDS).

INDICATOR NAME:	NIDS VERSION	FACTOR:	DEFINITION:	USE BY THE DEPARTMENT OF HEALTH
TOTAL NUMBER OF HIV TESTS CONDUCTED	2017 and 2020	No.	The total number of HIV tests conducted in all age groups.^2^	‘Monitors the impact of the pandemic and assists in better planning for effective combating of HIV and AIDS and decreasing the burden of diseases from TB’.
HIV TESTING RATE	2010	%	The number of clients tested for HIV, as a proportion of the number of clients counselled prior to HIV testing. Antenatal clients are excluded from this indicator.	Assesses the acceptability and uptake of HIV testing by clients visiting the public health facilities (Department of Health, 2019). It is also used as proxy to determine the extent to which the first 90-90-90 target (of 90% of people living with HIV aware of their status) is being achieved (Department of Health, 2019).
HIV POSITIVITY RATE (PREVALENCE IN DHIS) AMONG CLIENTS TESTED	2010	%	The proportion of clients that underwent HIV testing and were found to be HIV positive. This definition excludes antenatal clients.	
